# RNA-Sequencing and Bioinformatics Analysis of Long Noncoding RNAs and mRNAs in the Prefrontal Cortex of Mice Following Repeated Social Defeat Stress

**DOI:** 10.1155/2019/7505260

**Published:** 2019-03-27

**Authors:** Xian Wang, Shaolei Ma, Mao Mao, Caijuan Li, Xiaofeng Shen, Shiqin Xu, Jianjun Yang

**Affiliations:** ^1^Department of Anesthesiology, Obstetrics and Gynecology Hospital Affiliated to Nanjing Medical University, Nanjing, China; ^2^Department of Emergency and Critical Care Medicine, Zhongda Hospital Affiliated to Southeast University, Nanjing, China; ^3^Department of Anesthesiology, The First Affiliated Hospital of Zhengzhou University, Zhengzhou, China; ^4^Department of Anesthesiology, Jinling Hospital Affiliated to Nanjing Medical University, Nanjing, China

## Abstract

**Background:**

Repeated or continuous chronic psychological stress may induce diverse neuropsychiatric disorders; however, the underlying mechanisms remain unclear. In this study, we explored the expression profiles of long noncoding RNAs (lncRNAs) and mRNAs, along with their biological function and regulatory network, in mice after repeated social defeat (RSD) stress to explore their potential involvement in the development of anxiety-like behaviors.

**Main Methods:**

RNA-sequencing was used to screen all differentially expressed (DE) lncRNAs and mRNAs between the RSD and control groups. Quantitative real-time polymerase chain reaction (qRT-PCR) was used for confirmation of the RNA-sequencing results. The function of DE lncRNAs was predicted by Gene Ontology (GO) enrichment and pathway analyses of target mRNAs. In addition, the functional regulatory network of the target mRNAs was constructed to reveal potential relationships between lncRNAs and their target genes with bioinformatics approaches.

**Key Findings:**

In mice experiencing RSD, 373 and 454 lncRNAs, along with 1142 and 654, mRNAs were significantly upregulated and downregulated, respectively. The detailed regulatory network included 126 eligible lncRNA-mRNA pairs. Among them, 14 genes such as* Arhgef1, Chchd2, Fam107a, Dlg1, Nova2, Dpf1*, and* Shank3 *involved in neurite growth, neural development, and synaptic plasticity were direct targets of the DE lncRNAs. qRT-PCR of four of the DE lncRNAs and mRNAs confirmed the reliability of RNA-sequencing. GO clustering analyses showed that the top enriched biological process, cellular component, and molecular function terms were synaptic transmission, neuron spine, and glutamate receptor binding, respectively. Further, the top three significant enriched pathways were synaptic adhesion-like molecule (SALM) protein interactions at the synapses, trafficking of *α*-amino-3-hydroxy-5-methyl-4-isoxazolepropionic acid (AMPA) receptors, as well as glutamate binding, activation of AMPA receptors, and synaptic plasticity.

**Significance:**

Hundreds of lncRNAs and mRNAs are dysregulated after RSD, and many of these lncRNAs might participate in the development of anxiety-like behaviors via multiple complex mechanisms such as target regulation. Available informatics evidence highlighted the likely role of synapse dysfunction and abnormal synaptic neurotransmission in these behaviors. Thus, our findings provide potential candidate biomarkers or intervention targets for chronic psychological stress-induced neuropsychiatric disorders.

## 1. Introduction

Psychosocial stress is a significant contributor to the development of mental disorders or behavioral abnormalities such as anxiety, depression, schizophrenia, drug abuse, fear memory formation, or posttraumatic stress disorder [1]. We [2] and others [3] previously reported that social defeat stress, as a model of chronic psychological stress, may significantly induce anxiety-like behaviors. It is estimated that up to approximately 30% of the population is likely to experience various degrees of psychosocial stress in their lifetime, inevitably jeopardizing mental health and leading to substantial social or economic burdens [4]. Therefore, understanding the pathogenesis underlying the transition from chronic stress to a neuropsychiatric disorder is of great clinical significance and could help to identify possible intervention targets.

Long noncoding RNAs (lncRNAs) are a type of nonprotein coding RNA with a length ranging from 200 nucleotides to several hundred kilobases and mainly play a role in the response to harmful external stimuli [5]. In particular, lncRNAs are highly expressed in the central nervous system with large copy numbers and broad diversity. Accumulating evidence suggests that lncRNAs are involved in various levels of regulation of gene expression, including transcription or posttranscription regulation and epigenetic modification [6]. Notably, individual lncRNAs may have various functions, and such complex roles can orchestrate a combination of diverse biological processes.

Related to this high diversity in the central nervous system, animal model and human studies have suggested the participation of specific lncRNAs in multiple neuropsychiatric disorders, including anxiety, depression [7], and schizophrenia [8], via regulating neurodevelopment, neurodegeneration, and neuroimmunity. Certain lncRNAs have even emerged as candidate biomarkers for disease diagnosis, therapy, or prognosis [9]. However, the differential expression sites, binding sites, acting modes, and complex network of lncRNAs, which involve mRNAs, microRNAs,* cis* regulatory elements, and competing endogenous RNA networks, make it difficult to elucidate the specific functions of most lncRNAs [10].

Therefore, in the present study, we sought to investigate the roles of lncRNAs in the mechanism by which repeated or continuous chronic psychological stress induces diverse neuropsychiatric disorders using a mouse model of repeated social defeat (RSD) stress, based on a resident and intruder paradigm. Despite the involvement of several brain regions after repeated stress exposure, such as the amygdala or hippocampus, we chose to focus on the prefrontal cortex (PFC), which is considered to be the most sensitive brain region to control fear and stress responses [11]. Even slight acute stress can lead to rapid and prominent injury of prefrontal cognitive ability, while long-term chronic stress may even change the architecture of the PFC dendrites and thus alter its functional connectivity to the rest of the brain [12]. After inducing anxiety-like behavior, a marker of successful model establishment, we examined differentially expressed (DE) lncRNAs and mRNAs in the PFC of the mice using an RNA-sequencing approach. We further confirmed the RNA-sequencing results with four of the identified DE lncRNAs and mRNAs using quantitative reverse transcription-polymerase chain reaction (qRT-PCR). Subsequently, we predicted the biological function of the DE lncRNAs target mRNAs with Gene Ontology (GO) and pathway analyses in the RSD mice, and further constructed systematic functional landscapes of the lncRNAs–mRNAs network with specific bioinformatics approaches. These results can provide new insights and potential intervention targets for the early intervention or treatment of social stress-induced neuropsychiatric disorders.

## 2. Materials and Methods

### 2.1. Animals

Male 6–8-week-old C57/BL6 mice (20–25 g) were used as the residents, and 12-month-old CD-1 mice (30–35 g, retired breeders) were used as the intruders for RSD model establishment; all mice were purchased from Charles River Laboratory (Beijing, China). The C57BL/6 mice were housed in cohorts of three and the CD-1 mice were maintained in individual cages under a 12-h light/dark cycle at 22–24°C with sufficient food and water. All mice were acclimated to the surroundings for 7 days before model establishment. All procedures were conducted after ethical approval of the Institutional Animal Care and Use Committee of Nanjing Medical University (Approval no. 1706017).

### 2.2. RSD Model Establishment

The RSD model was established according to a resident-intruder paradigm as described previously [13]. In detail, an intruder male CD-1 mouse was placed into the home cage of three resident C57BL/6 mice. During the exposure, we recorded the behaviors of the mice to ensure that the intruder CD-1 mouse was acting aggressively, such as exhibiting attack behaviors, within 5–10 minutes after contact, while the resident C57/BL6 mice were presenting typical submissive behaviors, including an upright posture, escape attempts, or crouching. If no attack behaviors were observed by the CD-1 mouse or if the resident C57/BL6 mice attacked the intruder, a different CD-1 mouse was introduced. Each bout of exposure lasted 2 hours and was conducted for six consecutive nights; all anxiety-like behaviors of the C57/BL6 mice were monitored throughout the experiment. As a control, mice in a separate cage and room were left undisturbed under the same condition (3 mice per cage) throughout the experimental period.

### 2.3. Elevated Plus Maze (EPM) Test

The RSD-induced behavioral changes in C57/BL6 mice were evaluated with an EPM test, as a well-proven animal model for anxiety-like behaviors detection based on the aversion that rodents have for open spaces [14]. In brief, the apparatus contained four arms (70 × 5 cm) in the shape of a cross placed at a height of 75 cm above the floor. At the beginning of each test, the mouse was set into the center of the maze facing an open arm, and allowed to explore the maze* ad libitum*. The test was performed in a quiet room with fixed brightness and the movement trajectory was recorded and analyzed using SuperMaze animal behavior analysis software (Xinxin Ltd., Shanghai, China). The total time spent in the open and closed arms was recorded for each mouse with a 5-minute duration. Either an increase in time spent in the closed arm or a decrease in time spent in the open arm is considered to indicate anxiety-like behavior.

### 2.4. Tissue Collection and RNA Isolation

Only mice that presented with anxiety-like behavior were subjected to RNA-sequencing and bioinformatics analysis. For PFC tissue collection, eligible C57/BL6 mice were immediately decapitated after the EPM test. The brains were removed and cut coronally at the caudal border of the olfactory tubercle to remove the PFC [15]. Total RNA was extracted with Trizol reagent (Invitrogen, Carlsbad, CA, USA). RNA purity was assessed on a NanoPhotometer® spectrophotometer (IMPLEN, CA, USA) and the concentration was measured with Qubit® RNA Assay Kit on a Qubit® 2.0 fluorometer (Life Technologies, CA, USA) according to the manufacturer's instructions. In addition, RNA integrity was assessed with RNA Nano 6000 Assay Kit (Agilent Technologies, Foster City, CA, USA).

### 2.5. Library Preparation for lncRNA Sequencing

For RNA sample preparations, 3 *μ*g RNA served as the input material for each sample. Sequencing libraries were obtained using NEBNext® Ultra™ Directional RNA Library Prep Kit for Illumina® (NEB, USA) according to the manufacturer's protocol with index codes supplemented to generate sequences for each sample. In brief, ribosomal RNA was depleted with residues randomly cut into short fragments. First-strand cDNA was produced via a random hexamer primer, and the second strand was synthesized using DNA Polymerase I and RNase H. dNTPs with dTTP were substituted by dUTP in the reaction buffer. The left overhangs were changed into blunt ends with exonuclease and polymerase. Following adenylation of DNA 3′-ends and hairpin loop structure ligation, the library fragments were purified to select desired cDNA fragments of 150–200 bp. Next, 3 *μ*l USER Enzyme (NEB, USA) was mixed with the resultant cDNA and incubated at 37°C for 15 min followed by 95°C for 5 min. Thereafter, PCR was conducted with Phusion High-Fidelity DNA polymerase, Universal PCR primers, and Index (X) Primer. Finally, the products were purified with the AMPure XP system and the library quality was assessed (Agilent Bioanalyzer 2100 system). The generated libraries were sequenced by KeyGEN BioTECH (Nanjing, China) on an Illumina Hiseq 2500 platform, and 100-bp paired-end reads were produced. There are three samples obtained from three mice in each group, with data pooled for statistical analysis.

### 2.6. Clustering and Sequencing of lncRNA

Clustering of the index-coded samples was performed with a cBot Cluster Generation System according to the manufacturer's protocol. The library preparations were then sequenced with an Illumina Hiseq system and 150-bp paired-end reads were generated. We controlled the error rates of lncRNAs to be lower than 0.02% for each sample. The transcripts with splices for each sample were screened and combined as lncRNAs using Cuffmerge according to the following criteria: FPKM ≥ 0.5 (Cuffquant), exon number ≥ 2, and length > 200 bp. In addition, with Cuffcompare Software, we excluded overlapping and potential coding transcripts located in the exon regions [16]. All sequencing services were provided by KeyGEN BioTECH (Nanjing, China).

### 2.7. Identification of DE lncRNAs and mRNAs

We determined the DE lncRNAs and mRNAs using the edgeR package [17] along with the Cufflinks algorithm [18] among the transcripts obtained from RNA-sequencing. A |log_2_ fold change| ≥ 1.0 and p value ≤ 0.05 were used as the threshold values for identification of DE transcripts. Other than comparing candidate sequences with those of known lncRNAs with Cuffcompare [19], novel lncRNAs were identified according to the following criteria: (1) transcript length ≥ 200 bp and exon number ≥ 2; (2) a putative open reading frame < 300 bp; (3) overlapping prediction derived from the coding potential calculator, protein family database [20], and coding-noncoding index (CNCI) [21] related to possible lncRNA; and (4) not matched with known lncRNAs.

### 2.8. qRT-PCR Validation of DE lncRNAs and mRNAs

Four of the DE lncRNAs and mRNAs were randomly chosen for validation of the RNA-sequencing results in RSD versus control mice using qRT-PCR. In brief, total RNA was obtained from liquid nitrogen-frozen PFC tissues. Aliquots of 1 mg RNA were reverse-transcribed and amplified with a real-time PCR machine (Opticon, MA, USA) according to the manufacturer's recommendations. Expression levels of each lncRNA and mRNA were quantitatively calculated as a fold change with the 2^−ΔΔCT^ method. The* Gapdh* gene served as an internal control. The primer sequences used in qPCR are shown in Table 1.

### 2.9. Functional Prediction of the Target mRNAs of DE lncRNAs in RSD Mice

Since the majority of lncRNAs identified to date have not yet been functionally described, information on their function largely depends on the function prediction of their target mRNAs. lncTar software was used to predict the target mRNAs of the DE lncRNAs. All of the candidate target genes were then screened one by one to explore their possible involvement in psychiatric disorders with Medline database (PUBMED). Further, GO (http://geneontology.org/) was used to interpret the possible functions by forming hierarchical categories based on the biological process, cellular component, and molecular function terms of the DE target genes. GO is a useful bioinformatics tool that unifies the genes and gene product characteristics across all species and serves a good predictor of gene function.

In addition, pathway analyses were performed to identify the significant pathways of the DE targets genes through the Reactome (https://www.reactome.org), Kyoto Encyclopedia of Genes and Genomes (KEGG; http://www.genome.jp/kegg/pathway.html), Protein ANalysis THrough Evolutionary Relationships (PANTHER; http://www.pantherdb.org) and BioCyc (http://biocyc.org) databases. Enrichment factor, gene number, and p values were used to evaluate the correlation between target genes and their predicted functions or pathways. A threshold of p < 0.05 was considered to indicate a significant correlation. Other than KEGG, which stores the higher-order functional information for genes, the Reactome, PANTHER, and BioCyc databases were further screened to systematically elucidate the possible pathways involved in the response to RSD.

### 2.10. Analysis of lncRNA-mRNA Regulatory Networks

The coexpression regulatory network was constructed to determine the correlated expressed genes and establish lncRNA-mRNA coexpression pairs. Pearson's correlation coefficient was used with a default filtering threshold set at 0.99 [22]; the corrected p value of the hypothesis test was determined with the Holm calculation, with a default filtering threshold set at 0.05. Correlations among similar types of RNAs were not considered. lncRNA target genes were predicted with lncTar software.

### 2.11. Statistical Analysis

Data are expressed as mean ± standard error (sem). Unpaired t-test with Welch's correction was performed for comparison between two groups, and p < 0.05 was considered statistically significant.

## 3. Results

### 3.1. RSD for Six Days Successfully Induced Anxiety-Like Behaviors in C57/BL6 Mice

**Figure 1:**
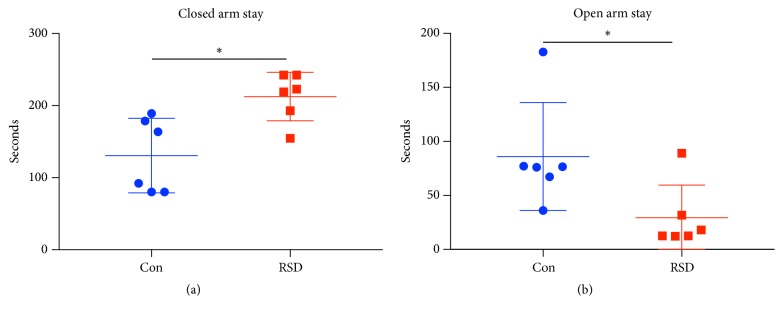
EPM test results for control or RSD stress C57/BL6 mice. Time spent in the open and closed arms is shown in (a) and (b), respectively. *∗* p < 0.05; N = 6 per group.

As there is individual variation among mice in response to chronic psychological stress, we examined anxiety-like behaviors in each mouse with the EPM test after six nights of exposure to social defeat stress. All six tested C57/BL6 mice demonstrated a significant increase in the time spent in the closed arm and a decrease in the time spent in the open arm compared to the controls (Figure 1), suggesting anxiety-like behaviors and successful establishment of the RSD model.

### 3.2. Hundreds of DE lncRNAs and mRNAs Were Identified in RSD Mice

**Figure 2:**
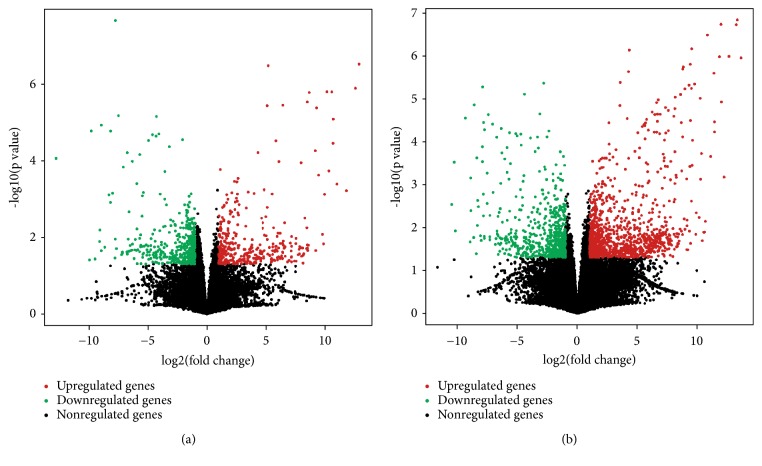
Volcano plot of DE lncRNAs (a) and mRNAs (b) in the PFC of RSD mice. Red and green dots indicate significant upregulated and downregulated genes (|log_2_ fold change| ≥ 1.0 and p value ≤ 0.05), respectively.

**Figure 3:**
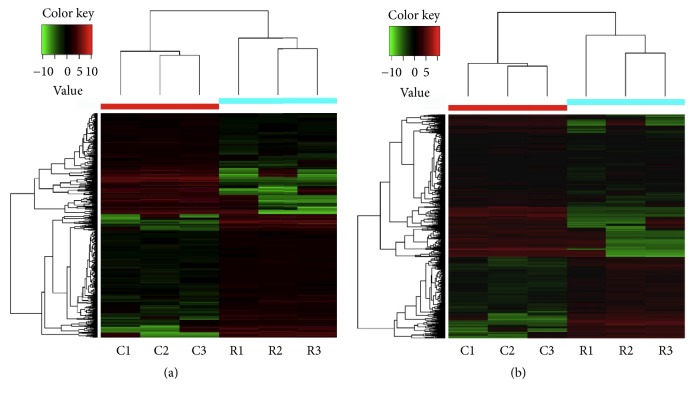
Heat maps of the hierarchical clustering analysis of overall DE lncRNAs (a) and mRNAs (b) in the PFC of RSD mice. Red and green lines indicate significantly upregulated and downregulated genes (|log_2_ fold change| ≥ 1.0 and p value ≤ 0.05), respectively.

RNA-sequencing revealed that 373 lncRNAs were prominently upregulated and 454 lncRNAs were downregulated in the PFC of RSD mice as compared to that of control C57/BL6 mice. In addition, 1142 and 654 mRNAs were significantly upregulated and downregulated, respectively, in the RSD mice. Data of all differential expressed lncRNAs and mRNAs have been uploaded to GEO database and released since March 5, 2019 (Accession number GSE127812). Tables 2 and 3 list the top 20 upregulated and downregulated lncRNAs and mRNAs in RSD mice, and the volcano plot and heat maps for clustering analysis of DE lncRNAs and mRNAs are shown in Figures 2 and 3, respectively. All differentially expressed lncRNAs and mRNAs, along with fold changes and p value were shown in Supplementary Tables 1–4.

### 3.3. qRT-PCR Validated the RNA-Sequencing Results

**Figure 4:**
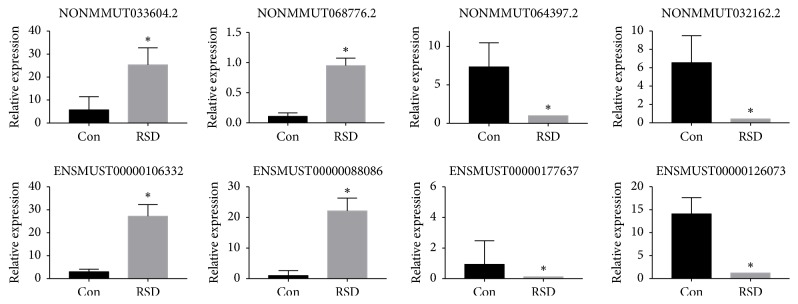
qRT-PCR results for four lncRNAs and four mRNAs in the PFC of RSD versus control mice. Each RNA sample was validated in triplicate, and the relative expression level of each gene was normalized to that of* Gapdh*. Data are expressed as mean ± sem; *∗* p < 0.05; N = 3 per group.

qRT-PCR showed that NONMMUT033604.2 and NONMMUT068776.2 were upregulated lncRNAs, and NONMMUT064397.2 and NONMMUT032162.2 were downregulated lncRNAs in RSD mice. In addition, ENSMUST00000106332 and ENSMUST00000088086 were upregulated mRNAs, and ENSMUST00000177637 and ENSMUST00000126073 were downregulated mRNAs in RSD mice. These results were consistent with the RNA-sequencing of these four genes (Figure 4), thereby validating the reliability of the RNA-sequencing protocol.

### 3.4. Target mRNA Prediction and Functional Regulatory Network of DE lncRNAs

**Figure 5:**
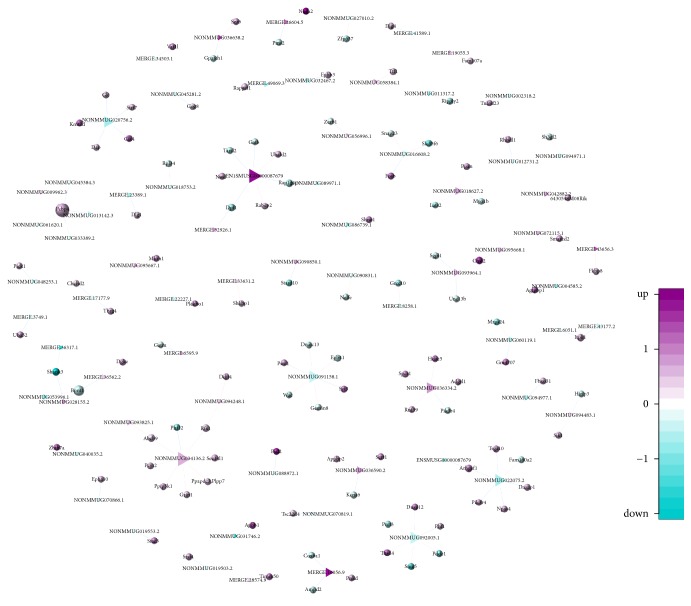
Regulatory network of lncRNAs and their target genes. Triangles and dots indicate lncRNA and mRNA symbols, respectively. Up- or downregulation and the associated fold change is indicated by the color gradient.

As lncRNAs may regulate adjacent protein-coding genes, including mRNAs, we further identified the likely target mRNAs of the identified DE lncRNAs using lncTar software, with a total of 126 target mRNAs determined. Unfortunately, no target mRNAs were identified for the top 20 upregulated and 20 downregulated DE lncRNAs (Table 2), which are more likely to play important roles in the pathogenesis in RSD mice. Indeed, a majority of the lncRNAs had no target mRNAs. Thereafter, we screened all possible target mRNAs according to the fold change in the expression level of two upregulated and two downregulated lncRNAs (Table 4). As shown in Table 4, one lncRNA could potentially target three or more mRNAs, associated with three or more regulatory functions. Thus, as expected, this result highlighted the complex regulatory network between lncRNAs and their target genes, as illustrated in Figure 5. Furthermore, a total of 14 promising target genes of the DE lncRNAs were identified to participate in the pathogenesis of certain psychiatric disorders (Table 5).

### 3.5. Functional Prediction of Target mRNAs of DE lncRNAs in RSD Mice

**Figure 6:**
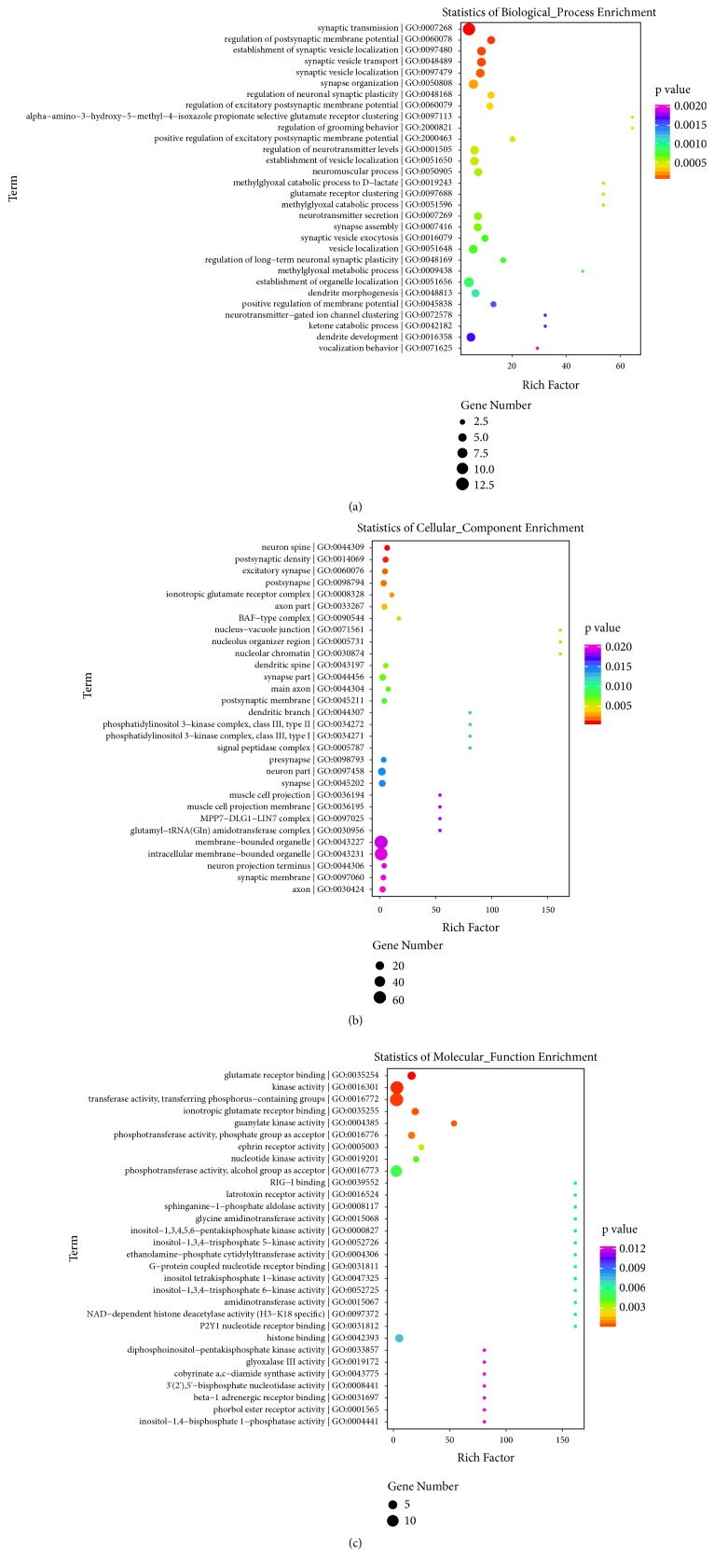
GO enrichment plot for lncRNAs target mRNAs. The top 30 enriched biological processes (a), cellular component (b), and molecular function (c) are presented according to gene number, p value, and enrichment factor. The horizontal axis represents the enrichment factor of the respective GO term, and the vertical axis indicates the enrichment terms, including their gene number and p value.

**Figure 7:**
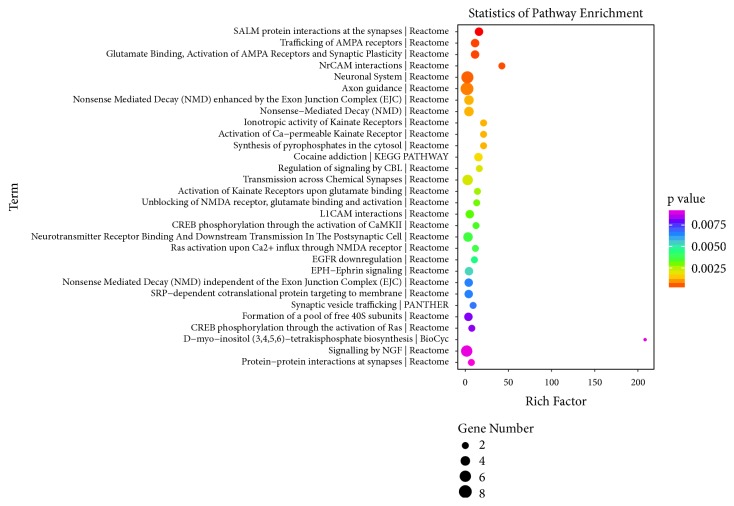
Pathway analysis for target mRNAs of DE lncRNAs in RSD mice. The top 30 enriched pathways are presented according to gene number, p value, and enrichment factor. The horizontal axis represents the enrichment factor of the respective pathway term, and the vertical axis indicates the enrichment terms, including their gene number and p value.

As shown in Figure 6, the top three significantly changed biological processes after RSD were found to be synaptic transmission, regulation of postsynaptic membrane potential, and establishment of synaptic vesicle localization. The top three enriched cellular components were neuron spine, postsynaptic density, and excitatory synapse, and the top three enriched molecular function terms were glutamate receptor binding, kinase activity, and transferase activity, transferring phosphorous-containing groups. These associations strongly indicate a common dysfunction of synapse in the PFC of RSD mice. The top three enriched pathways associated with the candidate target genes were synaptic adhesion-like molecule (SALM) protein interactions at the synapses, trafficking of *α*-amino-3-hydroxy-5-methyl-4-isoxazolepropionic acid receptor (AMPA) receptors, as well as glutamate binding, activation of AMPA receptors, and synaptic plasticity. The top 30 enriched pathway terms are shown in Figure 7.

## 4. Discussion

Social defeat is a well-validated model to mimic social stress in rodents [23]. In addition, RSD reproduces the pivotal behavioral, physiological, and immunological changes observed in humans that are subjected to chronic psychosocial stress [24]. As expected, six cycles of social defeat stress successfully induced anxiety-like behaviors in mice, as evidenced by a decrease in time spent in the open arm and in increase of time spent in the closed arm in the EPM test. Moreover, we identified hundreds of lncRNAs and mRNAs that are dysregulated in the mouse PFC after six cycles of social defeat stress, providing a first glimpse into the lncRNAs that might participate in the development of anxiety-like behaviors via multiple complex mechanisms via target regulation. The identified DE lncRNAs were scattered widely across all chromosomes other than sex chromosomes, although the majority are not annotated with available databases. In addition, four of the DE lncRNAs and four mRNAs were verified with qRT-PCR to confirm the reliability of the RNA-sequencing technique. Further, many of the candidate target genes of the DE lncRNAs, such as* Arhgef1, Chchd2, Fam107a, Dlg1, Nova2, Dpf1, *and* Shank3*, are involved in neurite growth, neural development, and synaptic plasticity. Furthermore, informatics analyses highlighted the contribution of synapse dysfunction and abnormal synaptic neurotransmission. Collectively, our findings provide potential candidate biomarkers or intervention targets for chronic psychological stress-induced neuropsychiatric disorders.

Accumulating evidence indicates that lncRNAs play important roles in diverse neuropsychiatric disorders. For example, the etiological lncRNA changes associated with major depressive disorder include the cognitive disorders-related lncRNAs* RNON, HAR1, PACER, *and* BACE1-AS*; synaptic plasticity-related lncRNAs* BDNF-AS, DISC2, *and* BC200;* or lncRNAs related to other psychiatric diseases such as* GOMAFU *and* MALAT-1* [23]. Among these, the lncRNA* GOMAFU*, which is related to schizophrenia, was previously shown to be significantly downregulated in mice with anxiety-like behaviors after fear conditioning [24]. The authors also suggested that* GOMAFU *interacts with the polycomb repressive complex1, BMI1, which regulates expression of the schizophrenia-related gene beta crystallin (*Crybbl*), thus playing a regulatory role in fear-induced anxiety-like behaviors.

Of note, most of the lncRNAs expressed in the mammalian brain are regionally enriched in the hippocampus and PFC, two brain regions with prominent roles in the pathogenesis of neuropsychiatric disorders [25]. Although we identified hundreds of DE lncRNAs and mRNAs in the PFC of RSD mice in our study, the majority could not be associated with a target gene. In fact, only nearly 70% of the lncRNAs in the PFC have been characterized in the mouse genome, and the functions of the majority of these lncRNAs are not well defined [25]. Thus, the unidentified lncRNAs warrant further exploration and might also have a relevant function. Overall, only 126 target mRNAs were identified for the DE lncRNAs identified in our study. Accumulating evidence suggests that lncRNAs not only function as enhancers or repressors to regulate gene expression but also act as molecular assemblers to participate in epigenetic modification [26]. Consistent with this idea, we found a complex regulatory network for lncRNAs and their target genes. For example, upregulated NONMMUG093964.1 can downregulate* Sgpl1* and* Unc13b* but can upregulate* Chst2*; simultaneously,* Chst2 *was found to be upregulated by another lncRNA, NONMMUG0956668.1.

Notably, when screening the function of the 126 target genes of the DE lncRNAs, 14 promising genes were found to participate in the pathogenesis of certain psychiatric disorders. For example,* Fam107a *(*Drr1*), the target gene of the lncRNA MERGE.19055.3, is recognized as acting as a unique link between stress and actin dynamics in the brain. FAM107A binds to actin, stabilizes actin filaments, facilitates bundling, and promotes actin-dependent neurite outgrowth, collectively regulating long-term potentiation and cognitive performance [27]. Increased expression levels of* Fam107a* were previously observed in the PFC, hypothalamus, hippocampus, and lateral septum in response to various types of acute social stress [28] or chronic stress such as social defeat or maternal deprivation [29]. Moreover, dysregulated* FAM107A* expression has been reported in postmortem PFC samples of suicide victims with schizophrenia and bipolar disorder [30]. In addition,* Shank3*, regulated by both MERGE.36317.1 and NONMMUG028155.2, encodes a widely expressed scaffolding protein (SHANK3) in the excitatory synapses, and its mutation leads to significant impairment of hyperpolarization activated cation channels, neuronal morphology, and synaptic connectivity [31].* Shank3* gene disruption could reproduce autism-like behaviors, including anxiety, social interaction deficits, and repetitive grooming behaviors, in mice [32], while* Shank3* overexpression could induce manic-like behaviors and seizures [33].* SHANK3 *polymorphisms have also been reported in individuals with autism [34], schizophrenia, and bipolar disorder [35]. In contrast to the commonly reported expression regions such as the hypothalamus, ventral tegmental area, and hippocampus, this is the first report demonstrating that the expression of* Shank3 *is also dysregulated after social defeat stress in the PFC. Other than these two genes, 18 other genes listed in Table 5 also appear to play potential roles in the development of behavioral abnormalities after RSD. Although their exact contribution is still far from clearly elucidated, our results highlight these genes as promising candidates for subsequent validation studies.

Moreover, GO enrichment analyses revealed that the significantly enriched biological process and cellular component terms related to the target genes of DE lncRNAs in RSD were mainly concentrated within the realm of synaptic functioning such as synaptic transmission, postsynaptic membrane potential regulation, and postsynaptic density. Further, the significantly enriched molecular function terms were glutamate receptor binding, kinase activity, or transferase activity, which are also involved in regulating synaptic plasticity and transmission. These findings are consistent with previous studies suggesting that synapse dysfunction such as synaptic formation [36], synaptic remodeling [37], and synaptic plasticity [38] plays a pivotal role in the pathogenesis of psychiatric disorders.

The significantly enriched pathways largely agreed with the GO results. The top three significantly enriched pathways of the DE genes were SALM protein interactions at the synapses, trafficking of AMPA receptors, as well as glutamate binding, activation of AMPA receptors, and synaptic plasticity. Indeed, SALMs, as a group of cell adhesion molecules, participate in regulation of neurite outgrowth, branching, synapse formation, and synaptic maturation [39]. In particular, SALM member 5 has been reported to be associated with severe progressive autism and familial schizophrenia, suggesting its clinical significance. In addition, dysfunction of AMPA receptors as well as the glutamatergic signaling pathway is well recognized to participate in the pathophysiology of psychiatric and neurological disorders [40]. Stein et al.[41] also suggested that synaptic connections damage due to microglia overactivation and the subsequent overproduction of proinflammatory mediators or cytotoxins significantly contributed to the development of social defeat stress-induced psychiatric disorders such as anxiety or depression. These data collectively indicate that maintenance of synapse function and normal neurotransmission may be an effective strategy for the intervention of psychiatric disorders. Nevertheless, several other GO or pathway terms could also be promising alternative directions for further research, and our findings only provide a preliminary exploration for detailed investigations in this regard.

Despite this potential, there are several limitations of this study that should be acknowledged. First, although use of an animal model is an advantageous tool to obtain valuable bioinformatics-based evidence, the results cannot be directly extrapolated to humans due to species differences. Second, other than the PFC, other brain regions such as the amygdala or hippocampus also play important roles in psychological stress-induced neuropsychiatric disorders, and deserve comprehensive exploration in the same regard. Finally, our results provide numerous candidate biomarkers or intervention targets; however, these candidates are only predicted using available informatics analyses or literature, and thus further research is required for validation of their specific roles.

## 5. Conclusion

We provide the first identification of DE lncRNAs and mRNAs in the mouse PFC after RSD stress using RNA-sequencing. Furthermore, bioinformatics analysis identified promising candidate target genes and the target regulatory network was constructed. These findings provide valuable insights into the molecular mechanisms underlying psychiatric disorders and also open up new possibilities for the development of novel therapeutic strategies for effective intervention.

## Figures and Tables

**Table 1:** Primer sequences of four changed lncRNAs and four mRNAs.

*lncRNA (5*′*-3*′)	Left Primer	Right Primer
NONMMUT033604.2	CCAGTTTCCCCAGCTTTTCC	GATGGCCTTTTCTGGTGCAA
NONMMUT068776.2	GAACGGCTTTCTTGGTTGGT	CAGAAAAGGAAAGACGGGGC
NONMMUT064397.2	TCCAGACCTCATTGTGCCAT	AAGTCATGGTGTCTGGTGGT
NONMMUT032162.2	GCAAAACCACGGTCTCTGTT	AAGGGAATGAGCAAAAGGGC

*mRNA (5*′*-3*′)	Forward Primer	Reverse Primer

ENSMUST00000106332	GGAGGATGAGATGATGCCAGA	GGTGGCTAGTGTGGGGTCT
ENSMUST00000088086	CTGCCACTATGGCTGCTGTC	GTTGGGCCGGATGTTCCTG
ENSMUST00000177637	GAGTATGACGATTCTGCTGAGG	CAGACCGAACGTGAAGACGAG
ENSMUST00000126073	GTTTCGACAGGGACGAAGTGT	TGGCGTCATACCCTCTAGCA

**Table 2:** The detailed information of the top 20 upregulated and 20 downregulated lncRNAs.

Gene ID	Log_2_ (fold change)	P value	Position	Track gene	Ensembl
*Upregulated*					

MERGE.45931.1	12.91088841	2.97701E-07	9:-:48651572-48787431	MERGE.45931	-
MERGE.29971.2	12.5979981	1.27501E-06	3:+:156929006-156954118	MERGE.29971	-
MERGE.19004.1	11.83667969	0.000600608	17:+:27655850-27684915	MERGE.19004	-
MERGE.14280.14	11.04431774	0.000406504	14:-:124199717-124259715	MERGE.14280	-
MERGE.30796.5	10.7196666	8.19282E-06	4:-:44126220-44233789	MERGE.30796	-
MERGE.38869.4	10.70506366	3.51044E-05	7:-:4628406-4640163	MERGE.38869	-
MERGE.15745.4	10.60865336	1.5956E-06	15:-:85535462-85581468	MERGE.15745	-
MERGE.20730.12	10.36177882	0.000183958	18:-:25477632-25753983	MERGE.20730	-
MERGE.1341.5	10.1919799	1.58113E-06	1:-:86350564-86358696	MERGE.1341	-
MERGE.27539.21	9.992578443	0.000754913	3:-:32954337-33143076	MERGE.27539	-
NONMMUT033604.2	9.91062089	0.014780123	19:-:5795745-5802674	MERGE.22023	NONMMUG020671.2
MERGE.22227.7	9.815364013	0.008371369	19:-:10245269-10304690	MERGE.22227	-
MERGE.28508.9	9.492582162	0.000238493	3:-:89234179-89246309	MERGE.28508	-
NONMMUT068776.2	9.321278483	4.16816E-06	9:-:43045840-43050998	MERGE.45767	NONMMUG042569.2
MERGE.25796.12	9.252992082	0.021990423	2:+:134786530-134876399	MERGE.25796	-
MERGE.25558.3	9.208027735	5.45061E-05	2:-:126628915-126675352	MERGE.25558	-
MERGE.31159.12	8.696361554	1.64867E-06	4:-:75944806-78211739	MERGE.31159	-
MERGE.11150.11	8.6314483	0.015990323	13:+:42766495-42853985	MERGE.11150	-
MERGE.43418.4	8.578314356	0.022050483	8:-:70778117-70805014	MERGE.43418	-
MERGE.6694.4	8.552744132	0.019202417	11:-:65002041-65162747	MERGE.6694	-

*Down-regulated*					

MERGE.45991.1	-9.94906637902031	0.038769757	9:-:52673044-52764976	MERGE.45991	-
MERGE.30595.2	-9.78735268889088	1.63371E-05	4:-:36853371-36863946	MERGE.30595	-
MERGE.42349.27	-9.47599858076888	0.036893626	8:-:17288423-17358373	MERGE.42349	-
MERGE.45094.8	-9.08839769230148	0.012891112	9:-:28996494-29475815	MERGE.45094	-
MERGE.35381.3	-9.06100615414186	0.006457129	5:-:125385977-125389800	MERGE.35381	-
MERGE.31194.1	-8.94438291137567	0.02225064	4:-:77979876-78060408	MERGE.31194	-
NONMMUT064397.2	-8.93847034165544	1.14889E-05	8:+:3455524-3456604	MERGE.42152	NONMMUG039739.2
MERGE.19402.3	-8.6496160845675	0.018028188	17:+:39843013-39848788	MERGE.19402	-
NONMMUT032162.2	-8.30049078658105	0.000779999	18:+:42333299-42341549	MERGE.21168	NONMMUG019797.2
NONMMUT061626.2	-8.16044749639102	0.001225066	7:-:59667720-59686523	MERGE.40818	NONMMUG038156.2
NONMMUT006358.2	-8.15305950707237	1.64402E-05	10:+:79767075-79769983	MERGE.4498	NONMMUG004092.2
MERGE.30727.11	-7.98663627685412	0.000701231	4:+:42670523-42875985	MERGE.30727	-
MERGE.5801.1	-7.78183232281183	0.034757085	11:-:20062304-20112909	MERGE.5801	-
MERGE.39926.3	-7.75105196885196	0.011104704	7:-:55973519-56019526	MERGE.39926	-
NONMMUT114518.1	-7.74074329257374	2.1705E-08	3:-:158067186-158076112	MERGE.29997	NONMMUG028350.2
NONMMUT065665.2	-7.66376363738363	0.019642797	8:+:55073249-55077138	NONMMUG040621.2	NONMMUG040621.2
NONMMUT002531.2	-7.4906570738083	6.51052E-06	1:+:130462576-130557075	NONMMUG001711.2	NONMMUG001711.2
MERGE.22685.10	-7.2275845681765	0.028413612	19:-:40292253-40513842	MERGE.22685	-
MERGE.26379.1	-7.15054223770817	0.024103564	2:+:160644759-160657971	MERGE.26379	-
MERGE.26349.9	-7.14898961475035	0.025448296	2:+:157424560-157432084	MERGE.26349	-

**Table 3:** The detailed information of the top 20 upregulated and 20 downregulated mRNAs.

Gene ID	Log_2_ (fold change)	P value	Position	Track gene	Symbol
*Upregulated*					

ENSMUST00000106332	13.64573771	1.10545E-06	7:+:126950966-126970270	MERGE.41644	Sez6l2
ENSMUST00000088086	13.33934534	1.42923E-07	2:-:166073920-166155285	MERGE.26566	Sulf2
ENSMUST00000018122	13.25066286	1.84766E-07	6:-:23262823-23839225	MERGE.36317	Cadps2
ENSMUST00000173888	12.64197878	1.0155E-06	5:-:134239513-134295608	MERGE.35535	Gtf2i
ENSMUST00000122356	12.23115962	0.000666844	5:+:81309912-81795216	MERGE.34310	Adgrl3
ENSMUST00000122037	12.0140956	1.16831E-05	5:+:81309912-81795216	MERGE.34310	Adgrl3
ENSMUST00000046463	11.97911042	1.82118E-07	15:+:79690895-79721478	MERGE.15503	Gtpbp1
ENSMUST00000060274	11.8574086	1.03636E-06	12:+:111166548-111267144	MERGE.10361	Traf3
ENSMUST00000197470	11.43826843	5.87443E-05	3:+:107101146-107107737	MERGE.29111	Kcna2
ENSMUST00000114573	11.43770849	3.40394E-05	9:-:45937875-45954977	MERGE.45887	Sidt2
ENSMUST00000038794	11.39272235	2.49989E-06	17:-:56186682-56218889	MERGE.19691	Dpp9
ENSMUST00000105661	11.12015081	0.000217624	4:+:152096719-152115390	MERGE.32825	Plekhg5
ENSMUST00000155907	10.85023001	3.19153E-07	15:-:94320334-94404258	ENSMUSG00000022449	Adamts20
ENSMUST00000107165	10.68586351	0.007139619	7:+:96211671-96908554	MERGE.40725	Tenm4
ENSMUST00000205303	10.60367115	0.012777534	7:+:56050877-56231132	MERGE.39933	Herc2
ENSMUST00000032220	10.54711543	0.012894258	6:-:124958816-124965448	MERGE.38366	Cops7a
ENSMUST00000085374	10.42208914	0.020121527	7:+:45163921-45176138	MERGE.39772	Slc17a7
ENSMUST00000119385	10.35553984	0.000186106	5:+:81309912-81795216	MERGE.34310	Adgrl3
ENSMUST00000109923	10.27873852	0.000746337	13:-:55473429-55488111	MERGE.11413	Dbn1
ENSMUST00000114499	10.2448629	9.59799E-06	X:+:73342621-73359080	MERGE.48255	Zfp275

*Downregulated*					

ENSMUST00000177637	-9.35288584494421	2.78E-05	9:-:119901616-120068283	MERGE.47489	Cx3cr1
ENSMUST00000126073	-8.95676408164527	0.021646	10:-:77290727-77418227	MERGE.4433	Adarb1
ENSMUST00000188674	-8.91289269577746	0.000705	1:-:71585523-71653172	MERGE.1078	Fn1
ENSMUST00000110688	-8.89118261770537	0.004053	8:+:22974844-23149933	MERGE.42453	Ank1
ENSMUST00000041838	-8.65979495858615	0.017237	1:-:25067678-25826760	MERGE.356	Adgrb3
ENSMUST00000108268	-8.61698882689041	1.36E-05	11:-:78962974-78984946	ENSMUSG00000001123	Lgals9
ENSMUST00000177432	-8.4434885275684	0.041071	18:+:35965067-36014715	MERGE.20997	Psd2
ENSMUST00000109081	-8.39951574161647	0.000233	11:+:52004221-52040593	MERGE.6361	Cdkl3
ENSMUST00000172142	-8.35069645803087	0.00902	14:-:31183313-31206826	MERGE.13022	Nisch
ENSMUST00000121927	-8.3287767124629	0.002357	16:-:76287400-76373827	MERGE.17718	Nrip1
ENSMUST00000070878	-8.26269044830275	0.003306	19:-:6977741-6980440	MERGE.22112	Fkbp2
ENSMUST00000110879	-8.09068328129774	0.015696	8:+:12915975-13019309	MERGE.42277	Mcf2l
ENSMUST00000103026	-8.03358200414137	0.020021	11:+:117115238-117157559	MERGE.8367	Sec14l1
ENSMUST00000179728	-7.93052673912365	0.000129	17:+:6978907-7011299	MERGE.18419	Rnaset2b
ENSMUST00000074989	-7.92684730635352	0.000286	9:+:44499136-44510388	MERGE.45814	Bcl9l
ENSMUST00000008826	-7.91029239938243	5.21E-06	X:+:74270812-74273135	MERGE.48303	Rpl10
ENSMUST00000120006	-7.85960187622417	0.001675	7:+:24112314-24127952	MERGE.39138	Zfp112
ENSMUST00000058041	-7.85110659309311	0.005348	9:-:15316917-15333499	MERGE.44892	Cep295
ENSMUST00000078200	-7.84051645619264	3.5E-05	7:+:141949751-141999005	MERGE.42082	Brsk2
ENSMUST00000091037	-7.78007365424463	0.016188	2:-:33406108-33428222	MERGE.23908	Zbtb34

**Table 4:** Detailed information of four significantly changed lncRNAs in RSD mice and their target mRNAs.

LncRNA Gene ID	lncRNA Symbol	mRNA Gene ID	mRNA Symbol	mRNA Gene Description
ENSMUST00000163836	ENSMUSG00000087679	ENSMUST00000057676	*Ubald2*	UBA-like domain containing 2
		ENSMUST00000126941	*Tarsl2*	-
		ENSMUST00000161254	*Nwd1*	NACHT and WD repeat domain containing 1
		ENSMUST00000128145	*Dpf1*	D4, zinc and double PHD fingers family 1
		ENSMUST00000151304	*Rabep2*	rabaptin, RAB GTPase binding effector protein 2
		ENSMUST00000139831	*Rap1gap*	-
		ENSMUST00000127348	*Gatb*	glutamyl-tRNA(Gln) amidotransferase, subunit B
MERGE.16056.9	MERGE.16056.9	ENSMUST00000106667	*Ampd2*	-
		ENSMUST00000138620	*Pnkd*	paroxysmal nonkinesigenic dyskinesia
		ENSMUST00000137766	*Cox6a1*	-
MERGE.36317.1	MERGE.36317.1	ENSMUST00000135214	*Shank3*	-
NONMMUT051222.2	NONMMUG031746.2	ENSMUST00000187057	*Apbb1*	amyloid beta (A4) precursor protein-binding, family B, member 1

**Table 5:** Fourteen candidate target genes associated with psychiatric disorders and their gene annotations.

lncRNA Gene ID	lncRNA Symbol	mRNA Gene ID	mRNA Symbol	Possible gene function in psychiatric disorders
ENSMUST00000133808	ENSMUSG00000087679	ENSMUST00000129928	*Arhgef1*	Expressed in cortical neural progenitor cells to regulate neurite outgrowth

MERGE.17177.9	MERGE.17177.9	ENSMUST00000094280	*Chchd2*	Response to mitochondrial stress; its mutation is reported in patients with Parkinson's disease

MERGE.19055.3	MERGE.19055.3	ENSMUST00000137133	*Fam107a*	A unique link between stress and actin dynamics, which regulates long-term potentiation and cognition performance

MERGE.23389.1	MERGE.23389.1	ENSMUST00000064477	*Dlg1*	A known brake on myelination in the central nervous system

MERGE.26604.5	MERGE.26604.5	ENSMUST00000172835	*Nova2*	Encodes NOVA2, a neuron specific RNA-binding protein, which regulates axon guidance during cortical development

MERGE.32926.1	MERGE.32926.1	ENSMUST00000128145	*Dpf1*	Essential for post-mitotic neural development and dendritic morphogenesis

MERGE.36317.1	MERGE.36317.1	ENSMUST00000135214	*Shank3*	Encodes postsynaptic scaffolding protein SHANK3, essential for hyperpolarization activated cation channels, neuronal morphology, and synaptic connectivity

MERGE.41589.1	ENSMUST00000135916	MERGE.41589.1	*Dlg4*	Encodes postsynaptic density 95 (PSD95), a major synaptic protein that clusters glutamate receptors and is crucial for synaptic plasticity

NONMMUT035884.2	NONMMUG022075.2	ENSMUST00000129928	*Arhgef1*	Same as aforementioned

NONMMUT035884.2	NONMMUG022075.2	ENSMUST00000056129	*Npas4*	As immediate-early gene, *Npas4* affects synaptic connection between excitatory and inhibitory neurons, neural circuit plasticity, and memory formation

NONMMUT045609.2	NONMMUG028155.2	ENSMUST00000135214	*Shank3*	Same as aforementioned

NONMMUT055012.2	NONMMUG034136.2	ENSMUST00000114317	*Grin1*	Encodes NMDA receptor GluN1 subunit; its mutation is reported in patients with epileptic encephalopathy, intellectual disability, and movement disorders

NONMMUT058932.2	NONMMUG036638.2	ENSMUST00000126866	*Sez6*	A candidate gene in autism, bipolar disorder, intellectual disability, and childhood onset schizophrenia

NONMMUT077752.1	NONMMUG048253.1	ENSMUST00000168513	*Pick1*	Encodes cytosolic protein PICK1, which facilitates the removal of GluA2 subunit from the synaptic plasma membrane

NONMMUT144756.1	NONMMUG091158.1	ENSMUST00000185503	*Dnajc13*	Dnajc13 mutation confers a toxic gain-of-function, impairs endosomal transport, and is an etiological contributor to Parkinson's disease

NONMMUT149941.1	NONMMUG094248.1	ENSMUST00000020308	*Ddit4*	Encodes DDIT4, a negative regulator of myelination during peripheral nerve system development

## Data Availability

The data used to support the findings of this study are available from the corresponding author upon request.

## Supplementary Materials

Supplementary Materials Table 1 presents the detailed information of all upregulated lncRNAs. Table 2 presents the detailed information of all downregulated lncRNAs. Table 3 presents the detailed information of all upregulated mRNAs. Table 4 presents the detailed information of all downregulated mRNAs.
